# The 9/3 Min Running Test: A Simple and Practical Approach to Estimate the Critical and Maximal Aerobic Power

**DOI:** 10.1002/ejsc.12254

**Published:** 2025-01-27

**Authors:** Santiago A. Ruiz‐Alias, Aitor Marcos‐Blanco, Iván Fernández‐Navarrete, Alejandro Pérez‐Castilla, Felipe García‐Pinillos

**Affiliations:** ^1^ Department of Physical Education and Sports Faculty of Sport Sciences University of Granada Granada Spain; ^2^ Sport and Health University Research Center (iMUDS) University of Granada Granada Spain; ^3^ Department of Education Faculty of Education Sciences University of Almería Almería Spain; ^4^ SPORT Research Group (CTS‐1024) CERNEP Research Center University of Almería Almería Spain; ^5^ Department of Physical Education Sports and Recreation. Universidad de La Frontera Temuco Chile

**Keywords:** modelling, running, severe intensity domain, training

## Abstract

This study aims to determine the validity of the linear critical power (CP) and Peronnet models to estimate the power output associated with the second ventilatory threshold (VT2) and the maximal aerobic power (MAP) using two‐time trials. Nineteen recreational runners (10 males and 9 females and maximum oxygen uptake: 53.0 ± 4.7 mL/kg/min) performed a graded exercise test (GXT) to determine the VT2 and MAP. On a second test, athletes performed two‐time trials of 9 and 3 min interspaced by 30 min. The CP was determined from the linear CP model and compared with the power output associated with the VT2. The MAP was determined from the linear Peronnet model, established at 7 min, and compared with the MAP determined in the GXT. The CP model was valid for determining the VT2, regardless of sex (*p* = 0.130; 9/3 vs. GXT: 3.5 [−1.1 to 8.2] W). The MAP was overestimated (*p* = 0.015) specifically in males (9/3 vs. GXT: 9.2 [3.3 to 15.1] W) rather than in females (*p* = 9/3 vs. GXT: 1.7 [−4.4 to 8.0] W). Therefore, MAP estimates were determined introducing the CP and W' parameters to a stepwise multiple linear regression analysis. For females, the CP was the unique significant predictor of MAP (*p* < 0.001) explaining 96.7% of the variance. In males, both CP and W' were significant predictors of MAP (*p* < 0.001) explaining 97.7% of the variance. Practitioners can validly estimate the VT2 and MAP through a practical testing protocol in both male and female recreational runners.

**Summary**

- Determining specific boundaries of the severe intensity domain is crucial for practitioners.
- The critical power concept enables the accurate determination of the lower boundary known as critical power. However, the definition of the maximal aerobic power, a key threshold within this domain, remains less explored.
- The linear transformation of the critical power and Peronnet models have been tested for this aim, resulting in an acceptable estimation of the second ventilatory threshold, and an overestimation of the maximal aerobic power, respectively.
- An adjusted equation with the critical power and work capacity over critical power has been proposed to determine the maximal aerobic power in recreational runners.

## 1 Introduction

Improving endurance performance is a complex mechanism that requires the correct application of different training principles (i.e., individualization, specificity, overload, progression, periodization, and reversibility) (Kasper 2019). On this basis, training periodization models pursued maximizing athletes' adaptions through different training intensities and volume distributions that confluence in its aim of improving the aerobic metabolism (Laursen 2010). Programming these periods involves the configuration of different training sessions (e.g., intervals, thresholds, and long runs) that require the accurate definition of specific training zones (Seiler and Tønnessen 2009).

As exercise intensity progresses, three intensity domains can be discerned according to the metabolic response (i.e., moderate, heavy, and severe) (Korzeniewski et al. 2022; Burnley et al. 2018). On the one hand, the moderate intensity domain is delimited in its upper limit by the first ventilatory threshold (VT1) or lactate threshold (LT) (Korzeniewski et al. 2022). The muscle damage from the accumulated volume could be established as the main cause of fatigue if substrate depletion is controlled (Burnley et al. 2018). Training sessions at these intensities, such as the so‐called easy runs (e.g., 60 min under LT) or long runs (e.g., 60 min at LT), pursued the accumulation of the overall weekly training volume (e.g., 80%) (Seiler et al.). On the other hand, the heavy intensity domain is defined by the intensity range between the VT1 and the second ventilatory threshold (VT2) (Korzeniewski et al. 2022). Glycogen depletion could be identified as the primary limiting factor within this intensity range (Burnley et al. 2018). Example of training sessions in this domain are the so‐called threshold or tempo run (e.g., 30 min at maximal lactate steady state). These conform part of specific training periods focused on maintaining the homeostasis regulation and accumulating medium‐to‐high training volumes (Issurin 2019). Lastly, the severe intensity domain is conceived by those range of intensities eliciting the maximal oxygen uptake ($\dot{V}O_{2}$_max_) (Korzeniewski et al. 2022). The progressive loss of homeostasis regulation (i.e., accumulation of P_i_, H^+^) determines fatigue in this intensity range (Burnley et al. 2018). Training sessions at this domain conform the so‐called high‐intensity interval training (HIIT) (e.g., 6 × 3 min at 95% VO_max_) characterizing the polarized training distribution commonly applied prior to the competing block (Seiler and Tønnessen 2009).

To define these intensity domains, the gold standard procedure to determine the VT1, VT2, and $\dot{V}O_{2}$_max_ landmarks is to perform a graded exercise test (GXT) monitored through a metabolic chart (Bentley, Newell, and Bishop 2007). However, considering the limited accessibility to this equipment, practitioners may opt for other practical options. In this regard, the maximal aerobic speed/power (i.e., the intensity eliciting the $\dot{V}O_{2}$_max_, which any further increase would be provided by the increase of anaerobic sources [MAS/MAP]) can be validly estimated through the last fully completed stage or the corrected factor according to the length of the uncompleted stage of the GXT (Uger et al. 1980; Pallarés et al. 2019). However, training zones established from a relative intensity of MAS/MAP do not account for the variability of the VTs position among individuals (Keir et al. 2022). Thus, practitioners may struggle to prescribe the adequate intensity for those focused on preserving the homeostasis regulation (i.e., VT2) (Jamnick et al. 2020). Therefore, it would be adequate to establish some alternatives to determine these landmarks without deviating from the practical need of testing.

In the endurance‐training context, a practical testing procedure to determine the maximal metabolic steady state has been consolidated through the so‐called critical speed/power (CS/CP) concept (A. M. Jones et al. 2019). This one establishes that this threshold can be validly estimated through the mathematical modeling of the intensity and time relationship of three to five maximal efforts lasting from 2 to 15 min (Hill 1993), which applied to the different hyperbolic and linear models available (Ruiz‐Alias et al. 2023a), the asymptote, slope, or interception (i.e., CP) target the VT2 intensity (A. M. Jones et al. 2019). In order to increase the feasibility of this testing procedure, practitioners may opt to simplify the model to a two‐point configuration using a linear CP model. In this regard, a single testing session composed of two time trials of 9 and 3 min interspaced by 30 min has been recently established as a valid two‐point configuration to determine CP in running (Ruiz‐Alias et al. 2022).

**FIGURE 1:**
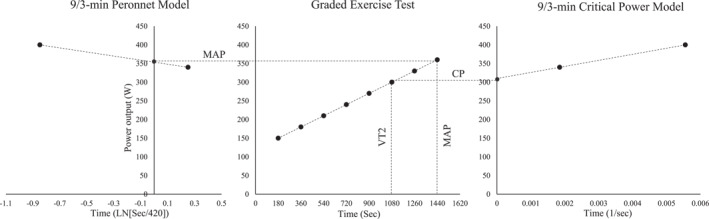
The illustration of the experimental design and modeling of the linear transformation of the Peronnet and critical power (CP) models to estimate the power output associated to the second ventilatory threshold (VT2) and the maximal aerobic power (MAP).

To avoid the need of performing two independent testing sessions to determine the MAS/MAP and CS/CP, the mathematical modeling of other models, such as the linear transformation of the Peronnet and Thibault model (Vandewalle 2017), offers the opportunity to use this two‐point configuration to estimate the MAP parameter. According to these authors, the fractional use of MAS/MAP can be traced through the slope of the relationship between the natural logarithm of the running duration with the running intensity being MAS/MAP established at 7 min (Peronnet et al. 1989). Therefore, the 9 and 3 min configuration would be an interesting testing procedure given that the linear CP (Whipp et al. 1982) and Peronnet models (Vandewalle 2017) would provide the CP and MAP estimates through the extrapolation and interpolation to the *Y*‐axis, respectively (Figure 1).

In addition to this novel approach for estimating the MAP, the validity of the 9 and 3 min configuration to determine the CP in running requires to be also tested in other populations and testing environments. In this regard, Ruiz‐Alias et al. (Ruiz‐Alias et al. 2022) provided valid CP estimates with respect to the VT2 obtained from a treadmill GXT protocol in highly trained male athletes. In other performance cohorts (e.g., recreationally trained), its validity requires to be also tested given that athletes might adopt different relative intensities to complete these fixed durations. In addition, the individuality of the VT2 location (Keir et al. 2022) also reinforces the need of testing the sensitivity of this two‐point model. Similarly, female athletes have been scarcely analyzed in this context (Ansdell et al. 2020; Bourgois et al. 2023; James et al. 2023). The existing studies determining the influence of sex on the CP in cycling revealed that its relative position concerning the MAP did not differ significantly between males and females (Ansdell et al. 2020; Bourgois et al. 2023), which requires to be explored in other disciplines such as running. Lastly, significant discrepancies have been reported between testing CP on the treadmill and track, which therefore different testing environments require to be considered (Ruiz‐Alias et al. 2023b). In order to fill these knowledge gaps, this study aims to determine the validity of the 9 and 3 min time trial configuration for estimating the VT2 and MAP using the linear CP and Peronnet models, respectively, in recreational male and female athletes on track.

## 2 Methods

### 2.1 Experimental Design

A repeated‐measures design was used to determine the validity of the VT2 and MAP estimates derived from the linear transformation of the CP and Peronnet models, respectively. In an outdoor track, subjects performed two testing sessions interspaced by 1 week. On the first, subjects completed a GXT composed of 3 min stages with increments of 1 km/h to determine the VT2 and MAP. On the second, subjects completed two time trials of 9 and 3 min interspaced by 30 min (Ruiz‐Alias et al. 2022). The CP was then determined from the linear CP model (Whipp et al. 1982) and compared with the power output associated with the VT2 (Figure 1) (Keir et al. 2024). The MAP was determined from the linear transformation of the Peronnet model (Vandewalle 2017) and compared with the MAP determined in the GXT. Testing sessions were preceded by two light training days and were performed under similar environmental conditions (temperature: 18°C–23°C; humidity: 30%–60%; and wind: < 10 km/h), time of the day (± 1 h), and with the same running shoes.

### 2.2 Subjects

Nineteen recreational runners (10 males and 9 females and age: 26 ± 5 years, height: 170 ± 10 cm, and body mass: 65 ± 11 kg) participated in the study. All subjects had at least 1 year of endurance training experience and no physical limitations that could compromise testing were reported (Table 1). All subjects were informed about the research purpose and procedures of the study before signing a written informed consent form. The study protocol adhered to the tenets of the Declaration of Helsinki and was approved by the institutional review board (No. 3274/CEIH/2023).

**TABLE 1:** Demographic, anthropometric, and performance measures from the graded exercise test and the two‐time trials.

	Females	Males	*p*‐value	ES
*n*	9	10	n/a	n/a
Age (years)	26.4 ± 4.5	26.6 ± 5.3	0.944	−0.04
Body height (cm)	162 ± 5	177 ± 8	0.000	−2.12
Body mass (kg)	55.8 ± 2.3	74.5 ± 8.8	0.000	−2.71
$\dot{V}O_{2}$_max_ (mL/kg/min)	53.0 ± 4.7	58.4 ± 5.3	0.032	−1.03
VT2 (% $\dot{V}O_{2}$_max_)	90.5 ± 8.5	91.7 ± 6.1	0.729	−0.16
MAP (W/kg)	4.1 ± 0.3	4.5 ± 0.2	0.005	−1.52
Time limit at MAP (min)	7.2 ± 2.9	9.3 ± 2.5	0.100	−0.74
CP (W/kg)	3.8 ± 0.3	4.2 ± 0.3	0.026	−1.27
W' (kJ)	6.4 ± 2.2	13.1 ± 4.0	0.001	−1.95
9 min (% CP)	105 ± 2	108 ± 2	0.040	−1.43
3 min (% CP)	117 ± 6	123 ± 7	0.040	−0.88

*Note:* Group mean ± standard deviation.

Abbreviations: $\dot{V}O_{2}$_max_: maximum oxygen uptake; CP: critical power; ES: Cohen's *d* effect size; MAP: maximum aerobic power; VT2: second ventilatory threshold; W': work over CP.

### 2.3 Power Meter

The Stryd power meter (Stryd Next Gen, Boulder, CO, USA) was used to determine the mean power output. This power meter version accounts for the power exerted to establish the running speed and to overcome wind resistance and gravity according to the body weight or gradient (Stryd; Cerezuela‐Espejo et al. 2020). Therefore, the body mass was measured with a weight scale (Seca 813; Seca Ltd, Hamburg, Germany) on the first testing session to set the power meter. For every testing session, the power meter was attached to the laces of the right footwear.

### 2.4 GXT

Subjects began the testing session with a standardized warm‐up, which consisted of 10 minutes of running at low intensity. After a series of dynamic mobility exercises, three progressive runs of 100 m with 2 minutes of rest were done to complete the warm‐up. The GXT comprised 3 min stages with speed increments of 1 km/h starting from 8 km/h (Bentley, Newell, and Bishop 2007). The pace was established by a researcher riding a bike close to the subject. The portable metabolic analyzer (COSMED K5, Rome, Italy) was used to determine the VT2, $\dot{V}O_{2}$_max_, and MAP, which was previously calibrated following the manufacturer's recommendations. The breath‐by‐breath data were examined to exclude errant breaths and those values outlying more than four standard deviations from the local mean were removed. Then, data were linearly interpolated to give 1 s values and then averaged into 10 s time bins. The smoothed breath‐by‐breath data were plotted against the power output to determine each physiological landmark. The VT2 was established as the power output at which the increase in VE/VO_2_ was followed by an increase in VE/VCO_2_ (Keir et al. 2022). The MAP was determined as the power output associated with the initial point of the plateau of the oxygen uptake. If this was not appreciable, the MAP was established at the stage where the subsequent increase was less than 2.1 mL/min/kg (Buchheit et al. 2013). The perceived effort reported on the Borg CR‐10 scale was used to determine the implication of the subjects.

### 2.5 9 and 3 min Time Trial

The subject began the testing session with the aforementioned warm‐up protocol. The 9 and 3 min time trial configuration was selected based on the validity of the CP estimates reported in highly trained athletes through this procedure (Ruiz‐Alias et al. 2022). Subjects were asked to complete the longest distance possible in each time trial. The 9 min time trial was performed first followed by the 3 min time trial after 30 min of rest. These were performed individually.

### 2.6 CP and Peronnet Linear Models

By means of a customized Excel spreadsheet, the power outputs recorded in the 9 and 3 min trials were plotted against the inverse of time to determine the CP as the interception of the regression line (Whipp et al. 1982):

$$\text{PO}=W^{´}*\left(1/t\right)+\text{CP}$$

where W' is the work over CP, PO is the absolute power output, and *t* is the time in seconds.

These were also plotted against the Naperian logarithm of the sustained time (i.e., 540 and 180 s) divided by the time limit at MAP proposed by Peronnet (i.e., 420 s) to determine the MAP as the interception of the regression line (Vandewalle 2017; Peronnet et al. 1989):

PO=MAP–E∗LN(t/420)

where *E* is the fractional use of MAP.

### 2.7 Statistical Analysis

Descriptive data are presented as mean ± SDs. The Shapiro–Wilk test confirmed the normal distribution of all variables (*p* > 0.05). A mixed model analysis of variance (ANOVA) was conducted on each parameter (i.e., CP and MAP) with the protocol (9/3‐min vs. GXT) as within‐subjects factor and sex as between‐subjects factor. Pairwise comparisons were identified using Bonferroni post hoc corrections. The magnitude of the differences between the CP and the VT2 was individually interpreted according to the smallest worthwhile change previously established (i.e., 5%) (Hill, Poole, and Smith 2002). After observing the bias reported by the Peronnet model, the time limit at MAP proposed by Peronnet of 420 s (Peronnet et al. 1989) was individually corrected by moving the *Y*‐axis to the MAP obtained in the GXT. An alternative procedure to estimate the MAP was determined by introducing the CP and W' parameters to a stepwise multiple linear regression analysis. All statistical analyses were performed using the software package SPSS (IBM SPSS version 25.0, Chicago, IL, USA) and statistical significance was set at an alpha level of 0.05.

## 3 Results

### 3.1 VT2 Estimation

**FIGURE 2:**
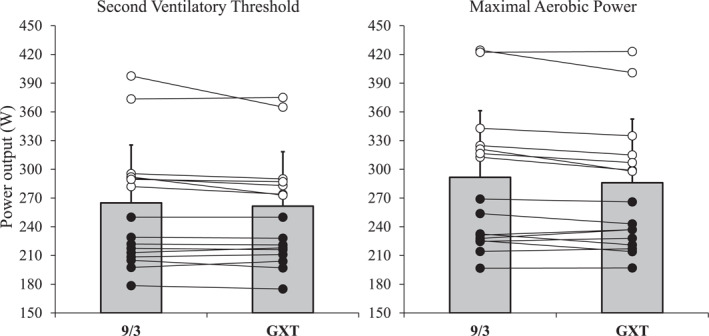
Power outputs associated with the second ventilatory threshold (VT2) and the maximal aerobic power (MAP) determined from the graded exercise test (GXT) and from the ones derived from the modeling of the 9 and 3 min time trial.

There was no significant main effect on the VT2 estimation (*p* = 0.130 and 9/3 vs. GXT: 3.5 [−1.1 to 8.2] W) (Table 2). There was no significant interaction between method and sex (*p* = 0.168), although a trend to overestimate the VT2 in males (9/3 vs. GXT: 6.7 [0.3 to 13.1] W) rather than in females (9/3 vs. GXT: 0.33 [‐6.4 to 7.1] W) was appreciable. However, the individual analysis revealed that 18 out of 19 subjects reported a VT2 estimate under the 5% validity threshold established (Figure 2). The average score on the CR‐10 scale was 9.0 (range 8–10) for the 9 and 3 min time trials and GXT sessions.

**TABLE 2:** Comparison between the power output associated to the second ventilatory threshold (VT2) and the maximal aerobic power (MAP) determined from the graded exercise test (GXT) with the ones derived from the modeling of the 9 and 3 min time trial.

Threshold	Sex	9/3	GXT	ANOVA
VT2 (Watts)	Males	312 ± 43.7	305 ± 40.7	Method: F_(1,17)_ = 2.537 and *p* = 0.130 Method × Sex = F_(1,17)_ = 2.079 and *p* = 0.168
	Females	214 ± 19.9	213 ± 20.8	
MAP (Watts)	Males	346 ± 47.1	337 ± 48.2	Method: F_(1,17)_ = 7 0.323 and *p* = 0.015 Method × Sex: F_(1,17)_ = 3.348 and *p* = 0.085
	Females	231 ± 20.9	229 ± 19.8	

*Note:* Data are presented as means ± standard deviations.

Abbreviations: ANOVA: analysis of variance; F: Snedecor's F.

### 3.2 MAP Estimation

**FIGURE 3:**
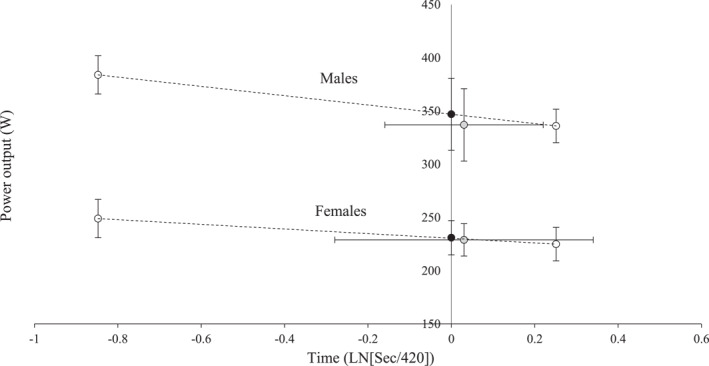
Comparison between the MAP (*y*‐axis) and its time limit (*x*‐axis) between the Peronnet model (black dot) and the ones obtained in the graded exercise test (grey dot). The 9 and 3 min power outputs are also illustrated (white dots).

There was a significant main effect on the MAP estimation (*p* = 0.015 and 9/3 vs. GXT: 5.5 [1.2 to 9.8] W) (Table 2). There was no significant interaction between method and sex (*p* = 0.085), although a trend to overestimate the MAP in males (9/3 vs. GXT: 9.2 [3.3 to 15.1] W) rather than in females (*p* = 9/3 vs. GXT: 1.7 [−4.4 to 8.0] W) was appreciable. Thus, the 420 s initially proposed by Peronnet was similar to the mean time limit at MAP observed in females (433 [300 to 566] seconds) but lower than the one reported in males (562 [455 to 670] seconds) (Figure 3).

**FIGURE 4:**
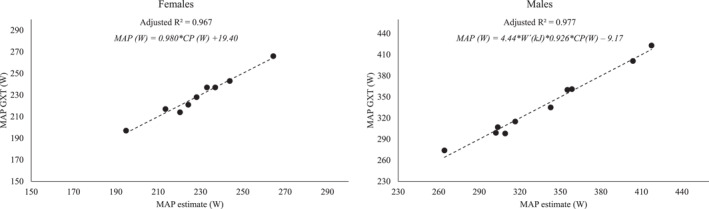
The level of agreement between the observed maximal aerobic power (MAP) values at the graded exercise test (GXT) and the ones obtained from the specific equations proposed for females and males using the critical power (CP) and the work over CP (W') as predictors.

The stepwise multiple linear regression analysis revealed that CP was the unique significant predictor of MAP in females (*p* < 0.001), explaining 96.7% of the variance. In males, both CP and W' were significant predictors of MAP (*p* < 0.001), explaining 97.7% of the variance. The resulted equations and level of agreements with the actual MAP values are displayed in Figure 4.

## 4 Discussion

This study aimed to determine the validity of the 9 and 3 min time trial configuration for estimating the VT2 and MAP using the linear CP and Peronnet models, respectively, in recreational male and female athletes. The results revealed that the two‐point configuration is a valid procedure to estimate the VT2 in recreational runners regardless of the sex. On the other hand, the MAP was overestimated through the linear Peronnet model, specifically, in males. Therefore, an alternative procedure to estimate the MAP using the CP and W' parameters is proposed, obtaining a high level of agreement with the actual values displayed in the GXT.

According to the results observed, practitioners can validly determine the intensity associated with the maximal metabolic steady state (A. M. Jones et al. 2019) through this configuration of two time trials of 9 and 3 min applied in a linear CP model. In conjunction to the results provided by (Ruiz‐Alias et al. 2022, 2024), it seems that this configuration is a valid procedure regardless of running performance (recreational vs. highly trained). It was initially hypothesized that the accuracy of the 9 and 3 min configuration to target the VT2 intensity through the *Y*‐intercept (i.e., CP) could have been compromised in athletes of different levels. These fixed durations would have been completed at different relative intensities, which in addition to the potential different VT2 location (Keir et al. 2022), other time trials durations could have been required. In this regard, (Ruiz‐Alias et al. 2022, 2024) reported that the highly trained athletes recruited completed the 9 and 3 min time trial at 105% and 115% of the CP. In the present study, although superior relative intensities were observed in the recreational male runners recruited (9 min: 108% CP and 3 min: 123% CP), it seems that both groups met the VT2 intensity through different slopes (i.e., W'). Lastly, in conjunction to the results provided by a recent study exploring the effect of the testing environment on the CP (treadmill vs. track) using the 9 and 3 min configuration (Ruiz‐Alias et al. 2023b), it should be noted that both conditions seem to be valid to determine the VT2 but not interchangeable.

Regarding the potential influence of sex on the CP estimation, the recreationally trained females recruited also displayed valid CP estimates through this procedure. The existing studies determining the influence of sex on the CP in cycling revealed that its relative position with respect to the MAP did not differ significantly between males and females (Ansdell et al. 2020; Bourgois et al. 2023). In line with the results here obtained, it seems that there is no need to adjust the testing procedure concerning sex. Similarly, it is worth noting that the females' power profile has been determined to be reproducible along the menstrual cycle in cycling (Bourgois et al. 2023). Therefore, extrapolating to the running field, it seems that these parameters could be valid to prescribe a full training cycle.

Regarding the MAP estimation, it is first necessary to clarify its concept in order to correctly interpret the results obtained. The MAP has been previously defined as the minimum intensity that would elicit the $\dot{V}O_{2}$_max_ (L. V. Billat et al. 1996), which actually corresponds to the intensity just above CP (L. V. Billat et al. 1996; A. Jones et al. 2011). At these intensities, the slow component of the oxygen uptake will increase at a certain rate (i.e., W') until reaching its maximal amplitude, that is, the $\dot{V}O_{2}$_max_ (A. Jones et al. 2011). Therefore, the MAP would be better defined as the intensity eliciting the $\dot{V}O_{2}$_max_, which any further increase would be provided by the increase of anaerobic sources (L. V. Billat et al. 1996). According to these definitions, the size of the over or underestimation would dictate the suitability of the testing procedure to prescribe the HIIT, which aims to accumulate the maximal time over 90% of $\dot{V}O_{2}$_max_ (Buchheit et al. 2013; Seiler et al. 2013).

It was determined that the linear Peronnet model overestimated the MAP, which could have therefore compromised the accumulable time of a HIIT session. Although the 7 min duration introduced in the model falls within the time limit at MAS/MAP reported in previous studies, ranging from 5 to 12 min (Berthon et al. 1997; Bertuzzi et al. 2012; Bellenger et al. 2015), this one is individual in nature (L. V. Billat et al. 1996) and irrespective of sex (V. Billat et al. 1966). Therefore, although the linear Peronnet model has shown to be valid in estimating long‐duration (i.e., 30 and 60 min) power outputs (Ruiz‐Alias et al. 2024, 2023c), its use is not recommended to estimate the MAP due to the error caused by the generic time limit proposed. In this regard, (Bertuzzi et al. 2012, 1997) determined that among different bioenergetics and neuromuscular variables that could determine the time limit at MAS in recreational long‐distance runners, the total energy production and the lower limb muscle power were the factors explaining the largest portion of variance (> 84%). These results converge with the role of the CP and W' parameters to discern the MAP among athletes. The relative aerobic contribution during the time limit at MAP was reported to be ∼83% of the overall energy production (Bertuzzi et al. 2012), being therefore the CP a good predictor. Regarding W', it is well known that this parameter is strongly correlated with the rate of the slow component of the oxygen uptake, reflecting therefore the time to reach the $\dot{V}O_{2}$_max_ (A. Jones et al. 2011). The cause of the different weighted roles of these parameters in each sex is unknown. L. V. Billat et al. (1996), (V. Billat et al. 1966) have also observed differences between male and female runners with respect to the physiological variables that determine performance in the severe intensity domain, being unaware of its possible cause, which therefore requires further exploration.

The novelty of monitoring the power metric in running should be also highlighted and discussed. The power meter here used has shown a high repeatability and level of agreement with the external work and oxygen uptake in different environments (i.e., indoor and outdoor) and conditions (i.e., speed, body weight, and slope) (Cerezuela‐Espejo et al. 2021; Imbach et al. 2020; Taboga et al. 2021). Therefore, it is not surprising that the CS and CP represent the same relative intensity on treadmill (Patoz et al. 2021). However, its utility with respect to monitoring the running speed is reinforced when different factors that could increase the external work arise, thus better reflecting the internal load. In this regard, (Borrani et al. 2003) reported that at a maximal effort at the severe intensity domain (i.e., 95% of MAS), the slow component of the oxygen uptake was accompanied by an increased vertical work. This observation could justify the results provided by (van Rassel et al. 2022), where the intensity associated with the maximal metabolic steady state was underestimated when monitoring the running speed, but not through the power metric, probably due to the misaligning caused by the slow component of the oxygen uptake, which was better capture through this latter.

Lastly, some limitations should be highlighted and discussed. Although VO₂max was determined by identifying the oxygen uptake plateau, a few athletes required the application of supplementary criteria (i.e., a subsequent increase of less than 2.1 mL/min/kg) to establish the MAP. Although subjective reports indicated maximal effort, incorporating verification bouts could have provided more robust the confirmation of MAP validity.

## 5 Conclusion

The 9 and 3 min time trial configuration applied to the linear CP model is a valid procedure for estimating the intensity associated with the maximal metabolic steady state in both recreationally trained male and female athletes. This configuration can be also applied to estimate the MAP using specific equations with the CP and W' parameters as predictors. The linear transformation of the Peronnet model significantly overestimated the MAP, specifically, in males and therefore, its use is not recommended for this aim.

## Conflicts of Interest

The authors declare no conflicts of interest.

## Practical Applications

Practitioners have available a valid and practical testing protocol to define the relevant boundaries of the severe intensity domain of their athletes. To facilitate this procedure, a supplementary file with the linear CP model has been created, where in addition to its associated CP and W' parameters, MAP estimates are provided according to the specific equations here displayed.

## Supporting information

Supporting Information S1
